# One-Step Quaternization/Hydroxypropylsulfonation to Improve Paste Stability, Adhesion, and Film Properties of Oxidized Starch

**DOI:** 10.3390/polym10101110

**Published:** 2018-10-08

**Authors:** Wei Li, Zhenzhen Xu, Zongqian Wang, Jian Xing

**Affiliations:** College of Textiles and Garments, Anhui Polytechnic University, Wuhu 241000, China; xuzhenzhen@ahpu.edu.cn (Z.X.); wzqian@ahpu.edu.cn (Z.W.); Xingjian@ahpu.edu.cn (J.X.)

**Keywords:** quaternized and hydroxypropylsulfonated, oxidized tapioca starch, quaternization/hydroxypropylsulfonation, paste stability, adhesion to fibers, film properties

## Abstract

To investigate the influences of quaternization/hydroxypropylsulfonation on viscosity stability, adhesion to fibers and film properties of oxidized tapioca starch (OTS) for ameliorating its end-use ability in applications such as warp-sizing and paper-making, a series of quaternized and hydroxypropylsulfonated OTS (QHOTS) samples were synthesized by simultaneous quaternization and hydroxypropylsulfonation of OTS with *N*-(3-chloro-2-hydroxypropyl) trimethylammonium chloride (CHPTAC) and 3-chloro-2-hydroxy-1-propanesulfonic acid sodium salt (CHPS-Na). The QHOTS granules were characterized by Fourier transform infra-red spectroscopic and scanning electron microscope techniques. Apparent viscosity and viscosity stability were determined, and adhesion was evaluated by measuring the bonding force of starch to the fibers. Film properties were also estimated in terms of tensile strength, breaking elongation, bending endurance, degree of crystallinity, and moisture regain. It was showed that quaternization/hydroxypropylsulfonation was capable of obviously improving viscosity stability of gelatinized OTS paste, enhancing bonding forces of OTS to cotton and polylactic acid (PLA) fibers, increasing breaking elongation, bending endurance and moisture regain of film and decreasing its tensile strength and degree of crystallinity, thereby obviously stabilizing paste viscosity, improving adhesion to fibers and lessening film brittleness. Increasing the level of quaternization/hydroxypropylsulfonation favored improvement in the stability, enhancement in adhesion and decrease in brittleness. The QHOTS showed potential in the applications of cotton and PLA sizing.

## 1. Introduction

As an abundant, inexpensive [1,2], renewable [3], and environmentally friendly polysaccharide material, starch has been widely applied either alone or in blends with other substances in the textile industry as a sizing agent and paper-making as a surface-sizing agent [4,5]. Starch possesses many anhydroglucose units (AGU) that include three hydroxyls and are linked together by α-d-glucosidic bonds [6]. Due to the sensitivities to heat [7], the bonds are easy to break and make apparent viscosity of cooked starch paste reduce over a period of time during sizing or coating. The decrease in viscosity leads to a decrease in starch add-on in sizing, causing a reduced weaving efficiency, or results in an instable surface-sizing, thereby affecting the paper quality. Consequently, starch paste must be stable in viscosity during its use. Usually, viscosity stability of the paste required is >90% during use [8]. Oxidized tapioca starch (OTS) is now applied in conventional size formulations for sizing natural and synthetic fiber yarns or surface-sizing in paper-making. Nevertheless, the viscosity of cooked OTS paste tends to reduce due to the breakup of d-glucosidic bonds during sizing. Therefore, OTS paste exhibits poor stability [9], adversely affecting the quality of products. Fortunately, cross-linking of granular starch with cross-linking reagents can stabilize paste viscosity of the starch. The desired effect has been achieved by reacting the starch with formaldehyde [10,11] or epichlorohydrin [12] or succinic acid [9] in an aqueous dispersion. Currently, the former two are generally applied for stabilizing the viscosity of cooked starch paste. However, formaldehyde can cause serious health problems [13], and epichlorohydrin is harmful to breathing organs and human eyes [14]. Accordingly, their uses in textiles should be controlled. Although cross-linking can stabilize viscosity, the intermolecular linkage introduced between starch molecules via cross-linking limits the thermal motion of starch segments [15] and affects water dispersibility of the paste, thereby generating a negative effect on adhesion to fibers and film properties of starch [9]. 

It has been confirmed that sufficient adhesion to fibers is perceived to be a valuable behavior and an essential character for starch used for sizing in textiles and paper-making [16]. In these applications, it functions as an adhesive to adhere the fibers together for strengthening fibrous goods such as papers and sized warps. Therefore, strong adhesion is often desirable since this produces an ameliorative end-use ability of starch in applications. In an application such as textile sizing, this requirement is extremely important because the adhesion, besides enhancing yarn strength, can lessen the hairs of warp yarns by gluing them back onto the body of yarns [11]. As a result, the adhesion is directly related to the physico-mechanical properties of sized warps [17]. As a result, adhesion is considered to be an extremely important index for estimating the quality of starch-based sizing materials. Film as a function of protecting the warps from mechanical abrasions can improve weaving ability [18]. In addition, film can enhance flexibility of paper. Therefore, flexible, extensible, and flex-fold resistant [19] film is particularly required. Obviously, the toughness of starch film occupies a very stringent place for the quality of fibrous goods. For these reasons, the adhesion behavior and the toughness of starch film have received increasing attention recently. 

**Scheme 1:**
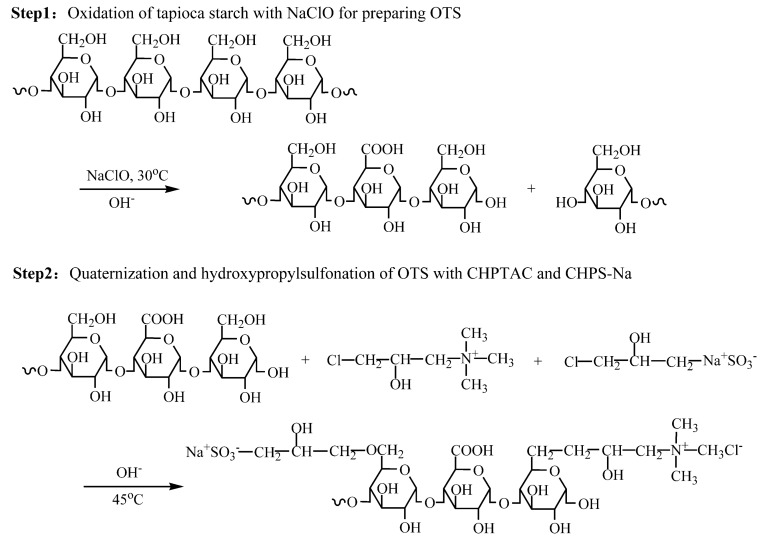
One-step quaternization and hydroxypropylsulfonation of oxidized tapioca starch (OTS) for the preparation of quaternized and hydroxypropylsulfonated OTS (QHOTS).

Anionic and cationic starches are the two types of chemically modified starches generally used in textile and paper-making. The modifications can promote the usefulness of starch for industrial applications [20]. However, anionic starch shows a negative zeta potential in water, the same as the surfaces of cellulose fibers such as cotton fibers in water [21]. It will unavoidably induce repulsion upon interface of starch layer-fiber and generate an adverse effect on the adhesion of starch to fibers. To eliminate this adverse effect, positively charged cationic starch becomes an important alternative to improve adhesion through electrostatic attraction at the interface between positive starch layers and cellulose fibers [22]. However, prior to dyeing, printing, and finishing, the starches sized onto warp yarns must be removed from the sized yarns. Attraction will inevitably increase the difficulty of the removal, bringing insufficient removal [23], and forming stains in fabric appearance [24]. In addition, it may induce serious problems such as paste flocculation, etc., during the use of cationic starch together with the negative auxiliaries in textile and paper-making industries [25]. Fortunately, one two-step method can be applied for introducing cationic and anionic groups into starch molecules [23]. Starch derivative containing negatively and positively charged groups simultaneously shows a possibility for overcoming the insufficiencies of cationic and anionic starches. The 3-(trimethylammonium chloride)-2-hydroxypropyl (TMACHP) and 3-propanesulfonic-2-hydroxy (PSH) groups as the cationic and anionic substituents, respectively, are capable of being derivatized into starch molecules by a one-step method, i.e., coinstantaneous quaternization and hydroxypropylsulfonation. The groups can exhibit strong steric restriction due to their large space volume. The restriction can serve as the wedges for disturbing the parallel arrangement of starch amyloses, and partly prevent the reassociation of starch hydroxyls during the formation of starch adhesive layers or starch films. Restriction [26] can play plasticization for layers or films, thereby reducing their internal stresses [27]. Therefore, quaternization/hydroxypropylsulfonation probably avoids the negative effect of cross-linking on adhesion to fibers and film properties of starch, as well as emphasizes that an improved adhesion of starch to fibers and a reduced brittleness of starch film may be expected, after the introduction of the two groups. Moreover, the introduced hydrophilic effect of TMACHP and PSH groups can enhance interaction and affinity of starch molecules with water and lower risk of degradation of starch macromolecules in aqueous paste. In addition, steric restriction mitigates the reassociation of starch hydroxyls [28] and retards ageing of the paste. Consequently, it can be expected to improve the stability of cooked OTS paste after the introduction of the groups. For these reasons, TMACHP and PSH groups were chosen and derivatized onto starch chains via double etherification, i.e., coinstantaneous quaternization and hydroxypropylsulfonation. The chemical structure of quaternized and hydroxypropylsulfonated OTS (QHOTS) is shown in Scheme 1. The preparation performed by the one-step method will shorten the reaction time and lessen the cost.

However, little was known about the influence on the properties of OTS (such as viscosity stability, adhesion, and film properties) after the introduction of positively charged TMACHP and negatively charged PSH groups because there was neither investigation on the impact of the quaternization and hydroxypropylsulfonation upon the properties nor was there evidence that revealed the properties could be improved by means of derivatization. Hence, one of the aims of this research is to reveal if the TMACHP and PSH groups can stabilize paste viscosity, enhance the adhesion to cotton fibers, and reduce the film brittleness of OTS. In addition, the influence of total degree of substitution (DS) on the properties was also analyzed since the usability of a modified starch depends on the amount of the groups introduced, i.e., DS [29]. Furthermore, polylactic acid (PLA) fibers as a new fiber material has good biodegradability and friendliness. Therefore, with increasing environmental pollution, it will be extremely important to improve its applications such as in textile and paper-making. As a result, another one of the aims is to determine if the QHOTS can enhance the adhesion to PLA fibers. The measurements were performed not only on QHOTS, but also on OTS. Such an experimental arrangement enabled us to obtain the impact of the groups themselves from the experimental data.

## 2. Experimental

### 2.1. Materials

Food-grade tapioca starch used as a raw material was obtained from Senhang Industrial Co., Ltd. (Shanghai, China). The 3-chloro-2-hydroxy-1-propanesulfonic acid sodium salt (CHPS-Na) was purchased from Jiaxing Sicheng Chemical Co. Ltd. (Jiaxing, China). *N*-(3-chloro-2-hydroxypropyl) trimethylammonium chloride (CHPTAC, 60% aqueous solution) used as received was an industrial product and supplied by Aladdin Industrial Corporation (Shanghai, China) and. The sodium hypochlorite (chemically pure) and other analytically pure chemicals (such as sodium hydroxide, anhydrous sodium sulfate, hydrochloric acid, methanol, and ethanol) were all obtained from Sinopharm Chemical Reagent Co. Ltd. (Shanghai, China). 

Pure cotton roving (460 tex) and PLA one (520 tex) applied for adhesion measurements, were kindly supplied by Talak Cotton Factory (Wuxi, China) and BBCA Group Co. Ltd. (Bengbu, China), respectively. 

### 2.2. Preparation of QHOTS

OTS applied for preparing the QHOTS was synthesized according to the literature [9]. 

The QHOTS was synthesized by simultaneously reacting OTS with CHPTAC and CHPS-Na in aqueous medium. Briefly, dried OTS (300 g) was suspended in distilled water containing Na_2_SO_4_ (30 g) for forming a 40% (*w*/*w*) starch aqueous suspension. The use of Na_2_SO_4_ was to prevent the swelling of starch granules. The suspension was transferred into a 1000 mL four-necked round-bottom flask. Then, the dispersion was adjusted to pH = 10–11 with a 3% (*w*/*w*) NaOH solution and heated to 45 °C in a water bath under continuous agitation. Afterwards, the aqueous solution of CHPTAC and CHPS-Na was added immediately into the suspension, and the suspension was adjusted to pH = 11–12.5 with the 3% NaOH solution. The reaction was carried out at 45 °C for 7 h under mechanical agitation. Finally, the rough product was neutralized to approximately pH 7 using dilute HCl solution, vacuum-filtered, washed with the mixture of ethanol and distilled water 3 times, vacuum-dried, pulverized, and sieved with a 100-mesh sieve.

### 2.3. Characterization of QHOTS

#### 2.3.1. Measurement of DS

DS value of starch can be described by the average number of substituents per AGU [30]. The DS_q_ was used to indicate the numbers of TMACHP groups introduced onto the starch chains, and was obtained by the calculation based on the nitrogen content from nitrogen analysis using the Kjeldahl method [31] with the following equation:

(1) $$DS_{q}=\frac{162(W_{1}-W_{0})}{1400-151.5(W_{1}-W_{0})}$$

where *W*_1_ (%) and *W*_0_ (%) denote nitrogen contents of QHOTS and OTS, respectively. 

DS_h_ denoted the numbers of PSH groups introduced onto the starch chains, and it was determined by the titration analysis [32] and calculated by the following equation:

(2) $$DS_{h}=\frac{162S}{3200-138S}$$

where *S* (%) and 138 denote the sulfur content and molecular weight of –CH_2_CH(OH)CH_2_SO_3_H minus that of hydrogen atom, respectively. 

#### 2.3.2. Fourier Transform Infra-Red (FTIR) Spectroscopy Analysis

The infra-red spectra of OTS and QHOTS samples was conducted on an IRPrestige-21 FTIR Spectrometer (Shimadzu Co. Ltd., Kyoto, Japan) to investigate the change in chemical structure of starch before and after quaternization/hydroxypropylsulfonation. Before the measurement, the powdered starch samples were blended with KBr, respectively, and then laminated. The spectra were recorded in a wave number range of 500 to 4000 cm^−1^. 

#### 2.3.3. Scanning Electron Microscopy (SEM)

An S-4800 scanning electron microscope (Hitachi Limited, Tokyo, Japan) was used to analyze the surface morphology of the OTS and QHOTS samples and observe the cross-section of the film samples. Before the determination, all the samples were coated with a thin layer of gold.

#### 2.3.4. Apparent Viscosity and Viscosity Stability

A 6% (*w*/*w*) aqueous suspension of the sample was prepared by suspending 24 g (dry base) of sample in 376 mL distilled water, and then heating it to 95 °C for 1 h under mechanical agitation in order to form a gelatinized starch paste. The apparent viscosity of the gelatinized starch paste was determined with an NDJ-79 Rotary Viscometer (Tongji Electrical Machinery Plant, Shanghai, China) under a concentration of 6% (*w*/*w*) at 95 °C with shear rates of 2028 s^−1^ (10 mPa·s ≤ viscosity < 100 mPa·s), and 344 s^−1^ (viscosity reading ≥ 100 mPa·s), respectively [33]. The viscosity values reported were the averages of two individual tests for each case. 

Viscosity stability was tested according to the literature [34]. Briefly, the viscosity was determined every 0.5 h over a period time of up to 2 h after the starch had just been gelatinized for 1 h. The stability was calculated using the following equation:

(3) $$\mathrm{Viscosity}\;\mathrm{stability}\;(\%)=\left(1-\frac{V_{\max}-V_{\min}}{V}\right)\times 100$$

where *V*_max_ (mPa·s) and *V*_min_ (mPa·s) denote the maximum and minimum values of the viscosities recorded over the period, respectively; and *V* is apparent viscosity.

### 2.4. Adhesion to Fibers

A legal standard method (FZ/T 15001-2008) was employed to perform the adhesion measurement of starch to fibers. The method contains three steps, i.e., step 1: formation of completely gelatinized starch aqueous paste; step 2: impregnating the roving for 5 min using paste and drying the sized roving in air; and step 3: drawing test of sized roving. 

Briefly, 7 g (dry base) of sample material was suspended in 700 mL of distilled water. The suspension was thoroughly mixed using a mechanical stirrer and heated to 95 °C in a water bath for 1 h to form a 1% sample aqueous paste. Thereafter, the roving carefully wound onto a frame was impregnated with the paste for 5 min, and then air-dried at the room temperature. After being stored at 65% relative humidity (RH) and 20 °C for at least 24 h, the tensile force of the sized roving was determined on HD026NS Electronic Strength Tester (Hongda Experimental Instrument Co. Ltd., Jiangsu, China) with a clamping distance of 100 mm and at a drawing speed of 50 mm/min at 65% RH and 20 °C according to the literature [23]. In each case, the presented result was the average of 20 successful tests with which the abnormal values had been rejected, and tensile force was adopted to denote the adhesion of starch to fibers. 

### 2.5. Preparation and Measurement of the Films

A 6% (*w*/*w*) aqueous suspension of the sample was prepared and then heated to 95 °C for 1 h under mechanical agitation for forming the paste. Then, 400 mL of paste was cast on a smooth polyester film (650 mm in length × 400 mm in width) spread on a glass plate and dried at 20 °C and 65% RH for forming the film. Then, films were cut into strips (200 mm in length × 10 mm in width) and (100 mm in length × 5 mm in width), respectively.

The 200 mm × 10 mm strips were adopted to estimate tensile properties via determination of the tensile strength and breaking elongation of the films. Before being determined, the strips were maintained at 20 °C and 65% RH for 24 h. The determination was carried out on the Instron3365 Material Tester (Instron Co., Canton, MA, USA) with an initial chuck-distance of 100 mm and a drawing speed of 50 mm/min in accordance with the ASTM D 882-02 method. The data reported was the mean value of 20 samples obtained by measuring the samples.

The 100 mm × 5 mm strips were used to evaluate the bending endurance through measuring the value of cycles bent to break on an Y731 Tester (Changzhou Textile Instrument Plant, Jiangsu, China). The strips were determined at the same condition as measuring the tensile properties. The result reported was the average value of 10 successful tests with which the abnormal values had been rejected by statistical analysis.

The measurement of moisture regain of the film was performed by determining the weight loss of the film after drying at 105 °C in accordance with our previous report [8]. The measurement was conducted in duplicate per film sample, and the result was calculated using the equation:

(4) $$\mathrm{Moisture}\;\mathrm{regain}\;(\%)=\frac{w_{1}-w_{2}}{w_{2}}\times 100$$

where *w*_1_ and *w*_2_ denote the weights (g) of the films before and after oven-drying, respectively. 

The XRD-6000 X-ray Diffractometer (Shimadzu Co., Japan) with a wavelength of 0.154 nm CuKa radiation at generator settings of 40 kV and 30 mA was used to analyze the diffraction behavior of OTS and QHOTS films. The scanning region of diffraction were registered at Bragg angle (2*θ*) of 10° to 45° and at a scanning rate of 4°/min with an angular step of 0.02°. 

## 3. Results and Discussion

### 3.1. Characterization Analysis of QHOTS

**Figure 1:**
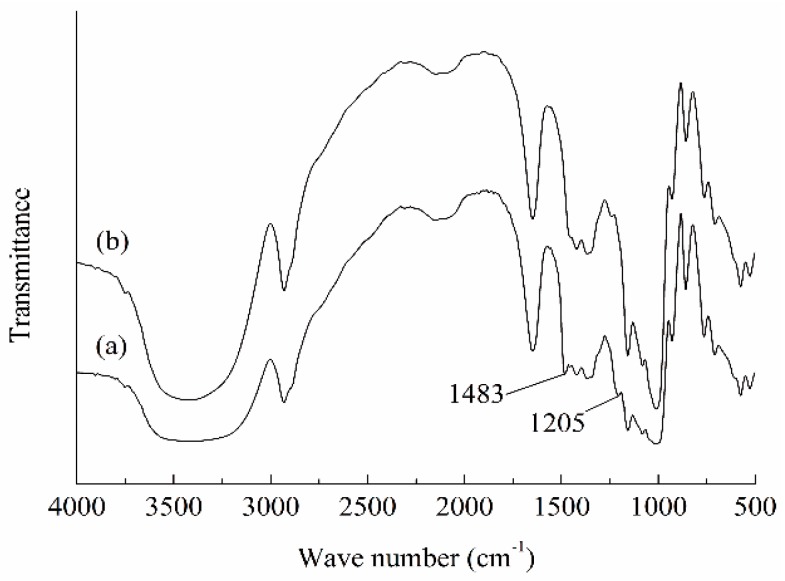
Fourier transform infra-red (FTIR) spectra of QHOTS (a), and OTS (b).

The introduction of TMACHP and PSH groups into starch molecules was confirmed by FTIR spectra of QHOTS (curve a) and OTS (curve b), as shown in Figure 1. Compared with the characteristic absorption peaks of OTS, there were two new absorption peaks in the spectrum of QHOTS. The new peak observed at 1483 cm^−1^ corresponded to the characteristic absorption band of C–N [35,36] in TMACHP groups, concluding the successful introduction of TMACHP groups on the starch chains. The other one appearing at 1205 cm^−1^ was attributed to the symmetric stretching vibration of sulfonic ions [37]. The peak denoted the existence of the PSH groups on the backbones of starch.

**Figure 2:**
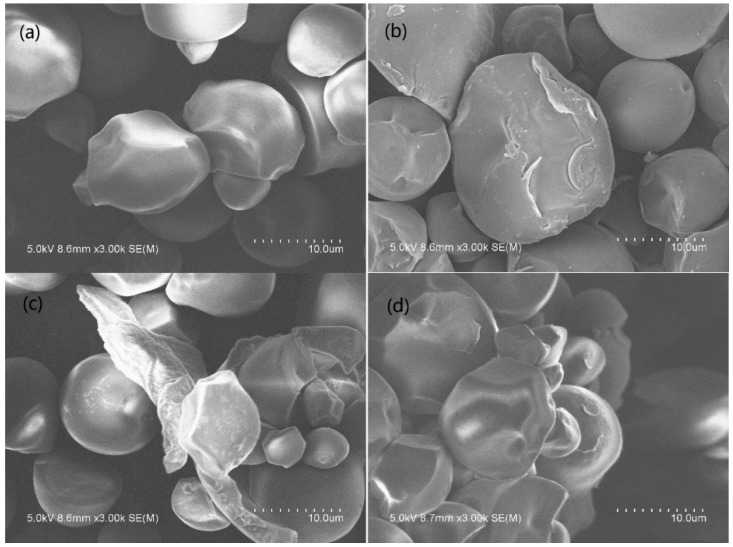
Scanning electron microscopy (SEM) images of OTS (**a**) and QHOTS with degree of substitution (DS) values of 0.014 (**b**), 0.026 (**c**) and 0.033 (**d**).

Starch modification can involve physical, chemical, and biochemical phenomena on the surface of the granules [38]. SEM has been described as an important means of clearly understanding the granular structure of modified starches and detecting most substituted regions in starch granules [34]. The effect of quaternization/hydroxypropylsulfonation on the surface morphology of starch granules is depicted in Figure 2a–d, respectively. It can be found from Figure 2a that the OTS granules have irregular, smooth surfaces, and polygonal shapes without visible fissures. Compared to OTS granules, QHOTS granules have some visible damage on the surfaces observed from Figure 2b–d. A probable explanation is that both quaternization and hydroxypropylsulfonation mainly occur on the surfaces of starch granules. In addition, the alkaline condition during the modification is likely to result in the change in the appearance of starch granules. 

**Figure 3:**
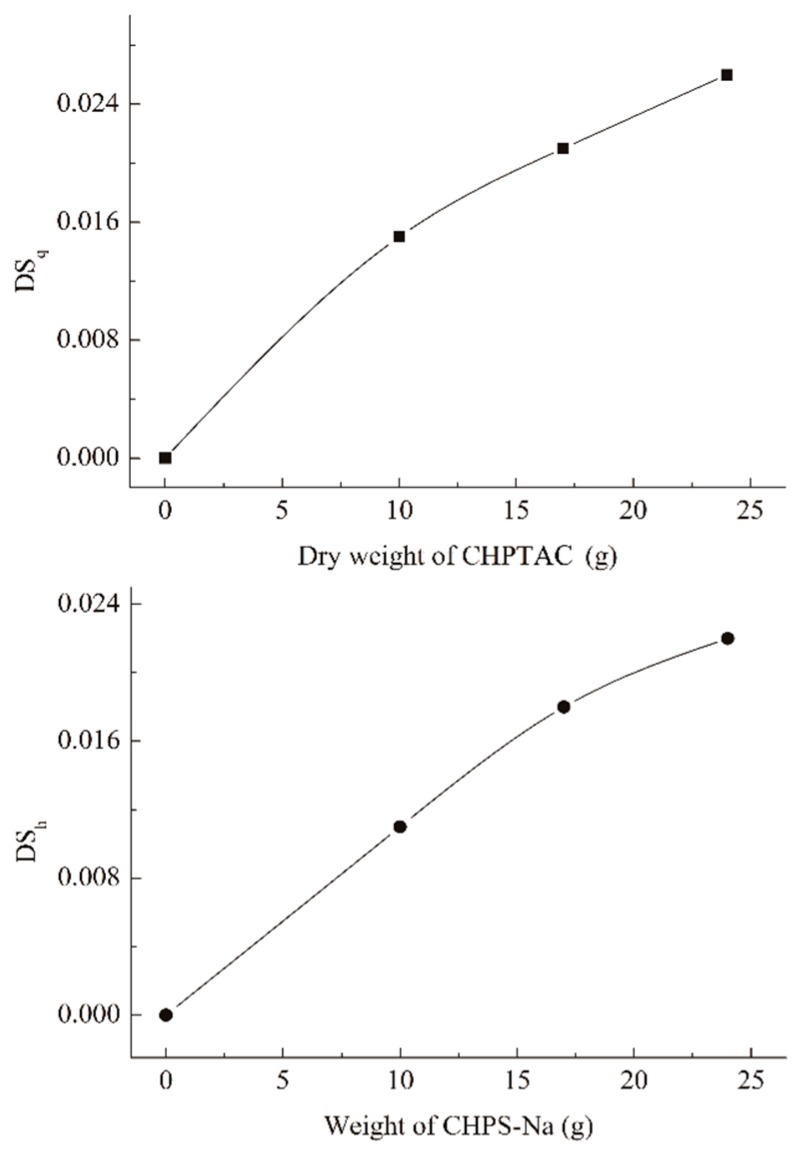
Degrees of substitution of starch modification, i.e., quaternization level (DS_q_) and the hydroxypropylsulfonation one (DS_h_) of OTS with *N*-(3-chloro-2-hydroxypropyl) trimethylammonium chloride (CHPTAC) and 3-chloro-2-hydroxy-1-propanesulfonic acid sodium salt (CHPS-Na), respectively.

The DS value can be considered as the average number of the substituted hydroxyl groups per anhydroglucose unit in the starch molecules by PSH and TMACHP. The DS (contains DS_q_ and DS_h_) values of granular QHOTS samples are measured, as illustrated in Figure 3. As expected, with the simultaneous increases in the amounts of CHPTAC and CHPS-Na to starch, the DS_q_ and DS_h_, i.e., the level of starch quaternization and that of the hydroxypropylsulfonation continuously rose. Accordingly, it can be imagined that QHOTS samples with gradually increased DS (the sum of DS_q_ and DS_h_) values can be obtained as the amounts raises.

### 3.2. Influence of Quaternization/Hydroxypropylsulfonation

#### 3.2.1. Influence on Apparent Viscosity and Its Stability

**Figure 4:**
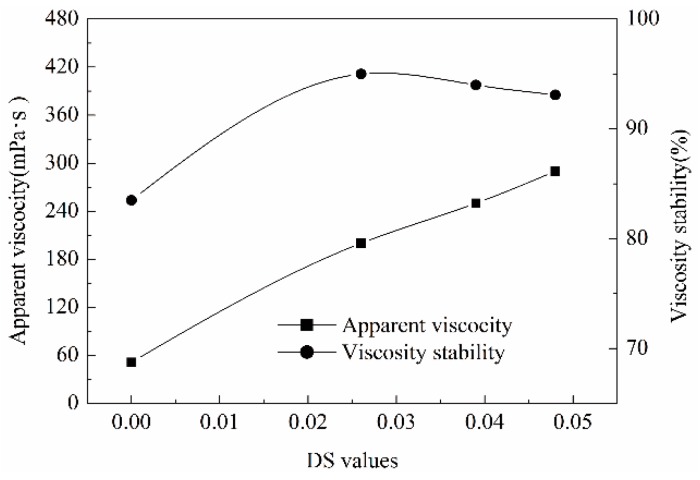
Apparent viscosity and its stability of gelatinized QHOTS paste.

The influence of the PSH and TMACHP groups introduced onto OTS chains on apparent viscosity and its stability of cooked OTS paste is shown in Figure 4. It could be seen that the groups increased the viscosity of cooked OTS paste. In addition, with the increases in DS values, i.e., the amounts of the PSH and TMACHP groups introduced, the viscosities gradually increased. Additionally, the QHOTS samples were superior to their counterpart OTS in the stability of paste viscosity. It is suggested that the introduction of TMACHP and PSH groups do not lead to a negative influence on the stability, meanwhile obviously improving the stability. 

Starch is a two-component carbohydrate polymer of linear polymer defined as amylose and branched polymer known as amylopectin [39]. Normally, due to the hydrogen bonding between the hydroxyls in the starch molecules, starch granules in water exhibit the characteristics of insolubility and structural integrity below its gelatinization temperature. However, starch granules in water at high temperature swell, and adsorb the water molecules, thus destroying the crystalline structure of starch to form amorphous structure, eventually forming a gelatinized starch paste [40]. The paste can be described as a biphasic system that is composed of a disperse phase described as swollen fragments mainly consisting of the amylopectin+ and a continuous one known as the amylose aqueous solution [41,42]. The TMACHP and PSH groups introduced are hydrophilic and ionizable and can promote the intermolecular interaction between starch and water and increase macromolecular distance through steric hindrance of the groups, thereby leading to greater expansion of soluble starch coils and further swelling of the fragments. The greater expansion and further swelling increase the kinetic volumes of the swollen fragments and coils, thereby resulting in an increase in the resistance to paste flow [26]. As a result, an increased viscosity is shown after the introduction of TMACHP and PSH groups into OTS molecules. The more the groups introduced, the greater the expansion and swellability of the starch in cooked paste. Consequently, as the DS increases, the viscosities show an ascending tendency. 

Viscosity stability of the paste influences the demand values of sizing pick-up during warp-sizing [11], and further exerts a certain influence on weavability. Therefore, starch paste must be stable in viscosity during warp-sizing. High stability is in favor of the stability of size pick-up [8], thereby promoting weaving efficiency. In addition, the stability in warp-sizing must be higher than 90%, with 85% for the inferior limit. As is well known, owing to the break of d-glucosidic bonds caused by their sensitivities to the heat and shear, degradation of macromolecular starch in cooked paste occurs easily, resulting in a fluctuation in viscosity [7] and producing low stability. For this reason, cooked OTS paste has an unstable viscosity, and the stability is less than 85%, as observed in Figure 4. However, the introduced hydrophilic TMACHP and PSH groups can ameliorate the paste stability. The main reasons are possibly due to the hydrophilicity and steric hindrance of the two groups. The hydrophilicity strengthens interaction and affinity between starch molecules and water and lowers the degradation of macromolecular starch in cooked aqueous paste. Besides, steric hindrance disturbs the formation of hydrogen bonds of starch hydroxyls and restrains paste ageing. As a result, the stability of cooked QHOTS paste at high temperature is significantly higher than that of the OTS paste.

#### 3.2.2. Influence on Adhesion

**Figure 5:**
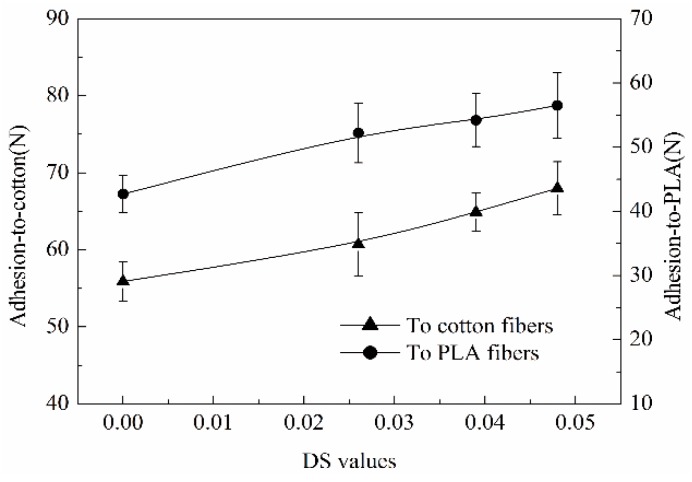
Influence of quaternization/hydroxypropylsulfonation on adhesion of OTS to cotton and polylactic acid (PLA) fibers, respectively.

It is well known that adhesion of sizing or coating agents to fibers exhibits the functions of enhancing the strength of fibrous goods by bonding the fibers together. Therefore, the influence of the chemical modification on adhesion should be evaluated. Figure 5 represents the influence of quaternization/hydroxypropylsulfonation on the adhesion of OTS to cotton and PLA fibers, respectively, and was evaluated as a function of the DS. It was observed that QHOTS was superior to OTS in the bonding forces to cotton and PLA roving. This suggests that quaternization/hydroxypropylsulfonation can raise the adhesion of OTS to cotton and PLA fibers. The forces of QHOTS depended on the total DS. As the DS increased from 0 to 0.048, the forces gradually increased. This implied that the increase in the DS helped to obtain higher bonding forces. 

To find the reasons for the increase in adhesion after quaternization/hydroxypropylsulfonation, the characteristics of the gelatinized starch paste must be given attention. Due to the association of hydroxyls between the macromolecules, the macromolecules of linear amyloses in low-temperature aqueous paste are inclined to orient themselves in a parallel fashion and form aggregates [43]. Furthermore, amylose molecules are also capable of co-crystallizing with the linear branches of amylopectin [44] in aqueous paste by the association of hydroxyls [45]. These can lead to paste retrogradation of starch and cause the microheterogeneity of the paste, inevitably exerting an adverse effect on the wetting and spreading of the paste onto the fiber surface [23]. The adverse effect can be described as incomplete wetting and outspreading. It has been proved that incomplete wetting and outspreading are harmful to adhesion capacity [46] owing to the interface failure that occurs around unwetted or outspread areas. Additionally, starch paste wets and spreads on the surface of fibers, shrinks in the volume due to the loss of water during drying, and converts into adhesive layers between the fibers in the roving. As the adhesive layer-fiber interfaces are fixed by adhesion, the shrinkage will induce strong internal stresses at the interfaces and within the matrix of the layers [23] due to the brittleness feature of starch. It has been suggested that the strong stresses can exert an intensive damage to the adhesion [46]. Undoubtedly, steric hindrance arisen from the TMACHP and PSH groups can disturb reassociation of starch hydroxyls and the regular arrangement of starch amyloses in the paste, thereby helping to alleviate paste retrogradation. Consequently, the TMACHP and PSH groups introduced are expected to ameliorate the wetting and spreading of the paste on the surface of fibers, thereby reducing the probability of interfacial failure that occurs around unwetted or outspread areas and resulting in an amelioration in adhesion. Besides, the hindrance of the groups can increase intermolecular distance between starch chains, and thus interfere with the hydrogen bonding of starch hydroxyls, promoting internal plasticization of the layers. Moreover, the hydrophilic TMACHP and PSH groups can absorb and store water within the matrices of the adhesive starch layers. This implies that the groups introduced can increase the water content of the layers. As is well known, water is an effective plasticizer for starch matrices [45]. Therefore, the introduced hydrophilic groups provide plasticization for the layers via raising the water content. As a result, the introduction of the groups favors an enhancement in the toughness of the adhesive layer between fibers, thus increasing adhesion. The disturbance in the reassociation and the plasticization generated are correlated with the amounts of the groups introduced, and with the increases in the amounts, the disturbance and plasticization gradually enhance. Accordingly, bonding forces of QHOTS to cotton and PLA fibers gradually increase, respectively, as the DS rises. 

#### 3.2.3. Influence on Film Properties

**Figure 6:**
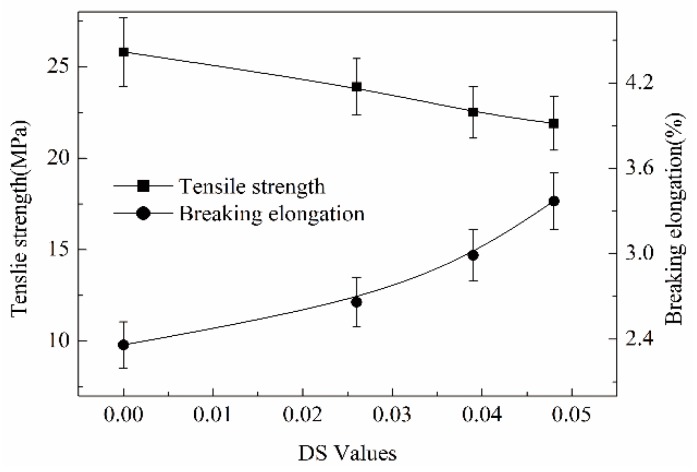
Influence of quaternization/hydroxypropylsulfonation on tensile properties of OTS films.

Figure 6 depicts the results of tensile properties such as tensile strength and breaking elongation of QHOTS films and OTS one (DS = 0, used as control). It could be seen that the films cast from cooked QHOTS pastes had greater breaking elongation and lower tensile strength than the OTS film. This means that introducing TMACHP and PSH groups into starch molecules can decrease the brittleness of the starch film. The maximal elongation (3.37%) of the QHOTS film was observed at the total DS value of 0.048 compared with the elongation of 2.36% for the OTS film. Moreover, breaking elongation and tensile strength of the QHOTS films depended on the total DS. With the rise in the DS, the elongation gradually rises and the strength reduces. There is no doubt that the QHOTS film with a higher elongation is preferable to the brittle OTS film for the applications in warp-sizing and surface coating. In addition, the thickness of OTS film was 0.121 mm, and the ones of the films of QHOTS with the DS values of 0.026, 0.039 and 0.048 were 0.124 mm, 0.122 mm and 0.127 mm, respectively.

**Figure 7:**
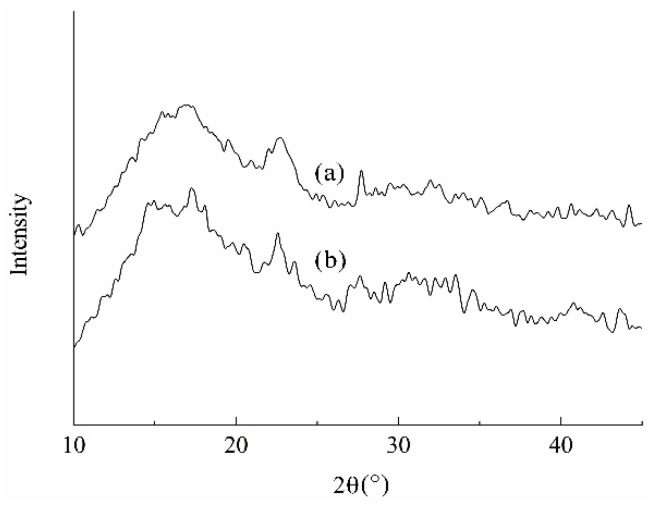
X-ray diffractograms of QHOTS film (a) and OTS one (b).

To understand the toughening effect of the TMACHP and PSH groups introduced on starch film, paste ageing, which is known as one of the major intrinsic shortcomings of starch, must be considered. During film formation, the amylose chains in aqueous paste re-associate and arrange parallel with each other by hydrogen bonds formed between the hydroxyls and form a three-dimensional network structure [47]. This is the major reason OTS film is brittle and cannot produce a large deformation. The strong steric hindrance shown due to large volumes of the TMACHP and PSH groups makes the groups act like the wedges between starch chains. For this reason, groups favor alleviation of the reassociation and decrease in the formation of intermolecular hydrogen bonds, thereby helping to disturb the arrangement of the amyloses in an orderly way during film formation. Accordingly, a decreased degree of crystallinity ascertained by X-ray diffraction of the starch film can be expected and is depicted Figure 7. Compared with an X-ray diffractogram of OTS film, the peak shape in that of the QHOTS film had switched from the spike to diffuse peaks. In addition, the QHOTS film was smaller than the OTS film in the areas of the crystalline regions observed from their X-ray diffractograms. It is suggested that the QHOTS film was lower than the OTS film in the crystallinity. The lower crystallinity is due to the disturbance in the regular arrangement of starch chains produced by the hindrance of the groups and decreases in the formation of the crystalline structure. Consequently, the QHOTS film exhibits a higher elongation and a lower strength by minimizing intermolecular hydrogen bonding. 

**Figure 8:**
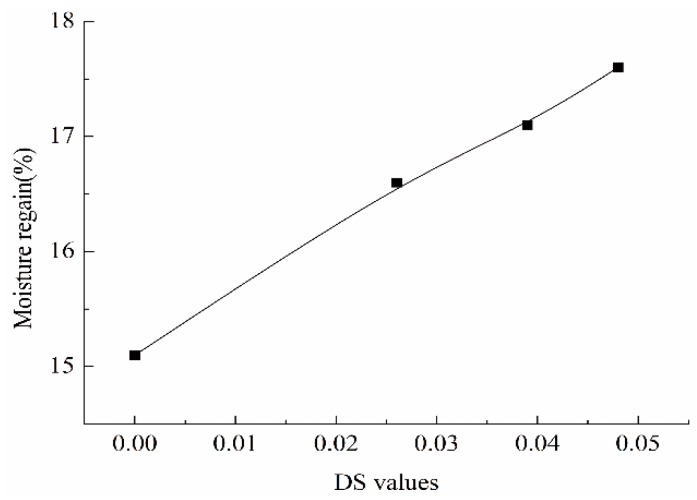
Influence of quaternization/hydroxypropylsulfonation on moisture regain of OTS films.

In addition, the hydrophilic groups introduced into the starch molecules can absorb water in the air. Water plays an important role in the tensile properties of starch film since it is an effective plasticizer for diminishing the brittleness of starch film [48,49]. Moisture regains of QHOTS films are determined as a function of their DS values, as represented in Figure 8. It could be found that the two groups evidently increased the regain of starch film, and the regain of QHOTS films was obviously more than that of the OTS one. This means that the groups introduced by starch quaternization/hydroxypropylsulfonation are capable of obviously increasing the regain of the film through absorbing water in air. The regain was correlated with the DS values, and it gradually increased as the values rose. The increase in the regain implies that the more water is kept within starch film. The water kept within starch film serves as a plasticizer that can promote the mobility of starch chains and diminish inter-chain hydrogen bonding [50], thereby enhancing the elongation of the film. In addition, the amount of water retained increases as the DS raises, leading to an obviously increased elongation. 

**Figure 9:**
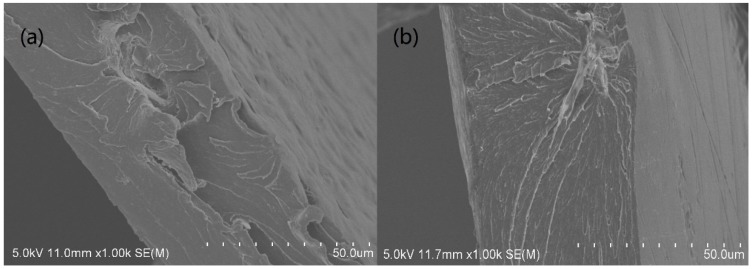
SEM images of the cross sections of OTS film (**a**) and QHOTS film (**b**).

**Figure 10:**
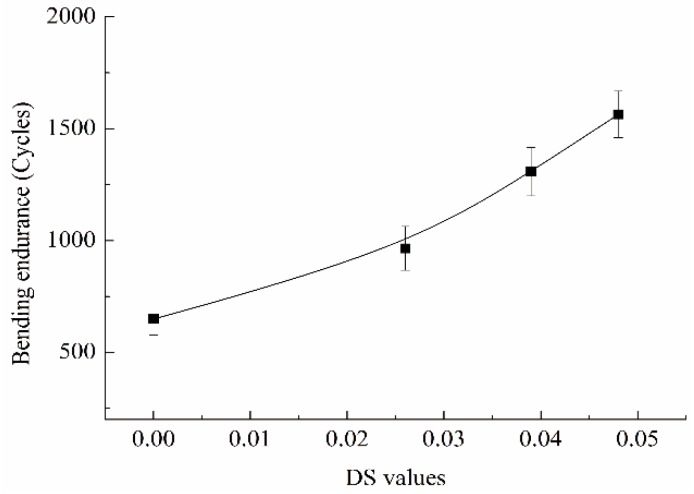
Influence of quaternization/hydroxypropylsulfonation on bending endurance of OTS films.

In the applications of warp-sizing and surface coating, a part of cooked starch paste is to form starch film around the surface of sized materials for making the materials sized possess good flexibility. However, the starch film such as OTS exhibits brittleness observed from the previous result and from the SEM image of its cross-section, as shown in Figure 9a. Therefore, the quaternization/hydroxypropylsulfonation of starch was performed for reducing its brittleness and enhancing its flexibility estimated by the investigation of bending endurance of the films, as shown in Figure 10. It could be seen that modification showed a positive effect on the endurance, and an increased endurance was obtained. Moreover, endurance presents noticeable correlation with the variation in the amounts of the two groups, and with the increases in the amounts, the number of bending cycles of QHOTS films gradually increases. This is attributed to the plasticization of starch film provided by the hindrance of the groups and the water absorbed due to their hydrophilicity. The plasticization increases the mobility of starch chains and reduces inter-chain hydrogen bonding [8], thereby decreasing internal stresses of starch films and strengthening bending endurance. Furthermore, as DS values increase, a consistently increased bending endurance is mainly due to the gradually enhanced plasticization. These data and Figure 9b indicate the importance of the modification in weakening film brittleness of OTS.

## 4. Conclusions

Based on this study, it could be concluded that introducing TMACHP and PSH groups into starch molecules via simultaneous quaternization and hydroxypropylsulfonation of OTS with CHPTAC and CHPS-Na was an effective way to overcome the drawbacks of OTS (instable viscosity, insufficient adhesion, and brittle film) for ameliorating its end-use ability in the applications such as warp-sizing and paper-making. Compared with 83.5% stability of OTS paste, the stability of QHOTS was higher than 90%, which indicated that derivatization was able to reduce fluctuation in viscosity and meet the need in the stability required in applications. The bonding forces of QHOTS to cotton and PLA fibers as well as breaking elongation, bending endurance and moisture regain of the film were superior to those of OTS, and tensile strength and degree of crystallinity of the film were inferior to those of OTS. These implied that derivatized TMACHP and PSH groups could improve the adhesion and reduction of the brittleness of film. In addition, the adhesion and film properties of QHOTS closely depended on total DS. Increasing DS led to gradually increased bonding forces, breaking elongation, bending endurance and moisture regain, and a consistently decreased tensile strength. Considering the drawbacks of OTS in paste stability, adhesion, and film properties, QHOTS with a DS of 0.048 showed potential in the applications of cotton and PLA sizing.
